# A Toll-like receptor identified in *Gigantidas platifrons* and its potential role in the immune recognition of endosymbiotic methane oxidation bacteria

**DOI:** 10.7717/peerj.11282

**Published:** 2021-04-30

**Authors:** Mengna Li, Hao Chen, Minxiao Wang, Zhaoshan Zhong, Hao Wang, Li Zhou, Huan Zhang, Chaolun Li

**Affiliations:** 1 Center of Deep Sea Research and Key Laboratory of Marine Ecology & Environmental Sciences (CODR and KLMEES), Institute of Oceanology, Chinese Academy of Sciences, Qingdao, China; 2 University of Chinese Academy of Sciences, Beijing, China; 3 CAS Key Laboratory of Marine Ecology and Environmental Sciences, Qingdao National Laboratory for Marine Science and Technology, Qingdao, China; 4 Center for Ocean Mega-Science, Chinese Academy of Sciences, Qingdao, China

**Keywords:** *Gigantidas platifrons*, Methane-oxidizing bacteria, Symbiosis, Immune recognition, Toll like receptor, Pattern recognition receptors

## Abstract

Symbiosis with chemosynthetic bacteria is an important ecological strategy for the deep-sea megafaunas including mollusks, tubeworms and crustacean to obtain nutrients in hydrothermal vents and cold seeps. How the megafaunas recognize symbionts and establish the symbiosis has attracted much attention. Bathymodiolinae mussels are endemic species in both hydrothermal vents and cold seeps while the immune recognition mechanism underlying the symbiosis is not well understood due to the nonculturable symbionts. In previous study, a lipopolysaccharide (LPS) pull-down assay was conducted in *Gigantidas platifrons* to screen the pattern recognition receptors potentially involved in the recognition of symbiotic methane-oxidizing bacteria (MOB). Consequently, a total of 208 proteins including GpTLR13 were identified. Here the molecular structure, expression pattern and immune function of GpTLR13 were further analyzed. It was found that GpTLR13 could bind intensively with the lipid A structure of LPS through surface plasmon resonance analysis. The expression alternations of GpTLR13 transcripts during a 28-day of symbiont-depletion assay were investigated by real-time qPCR. As a result, a robust decrease of GpTLR13 transcripts was observed accompanying with the loss of symbionts, implying its participation in symbiosis. In addition, GpTLR13 transcripts were found expressed exclusively in the bacteriocytes of gills of *G. platifrons* by in situ hybridization. It was therefore speculated that GpTLR13 may be involved in the immune recognition of symbiotic methane-oxidizing bacteria by specifically recognizing the lipid A structure of LPS. However, the interaction between GpTLR13 and symbiotic MOB was failed to be addressed due to the nonculturable symbionts. Nevertheless, the present result has provided with a promising candidate as well as a new approach for the identification of symbiont-related genes in Bathymodiolinae mussels.

## Introduction

Since their first discovery in 1977 and 1984, the hydrothermal vents and cold seeps have attracted a great of attention due to the chemosynthesis-driven ecosystems (Corliss et al., 1979; Kennicutt et al., 1985). Unlike the photosynthesis-driven ecosystems, chemosynthetic bacteria such as methane-oxidizing bacteria (MOB) and/or sulfide-oxidizing bacteria (SOB) are known as the primary producers and could fix the inorganic carbon into organic carbohydrates by oxidation of methane and hydrogen sulfide in the emitting fluids of hydrothermal vents and cold seeps. Besides the free-living MOB and SOB, megafauna such as mussels, galatheid crabs and tubeworms are also thriving in the chemosynthetic ecosystem (Danovaro et al., 2017). Moreover, it is found that some megafauna are in symbiosis with MOB and/or SOB and could obtain majority of their nutrition directly from them. In addition, some studies hypothesized that symbionts could even detoxify their host against high concentration of heavy metal and HS^-^ and protect hosts against parasites from the harsh environment (Jan et al., 2014; Sayavedra et al., 2015; Methou et al., 2019). The symbiosis between megafauna and chemotrophic bacteria are therefore suggested as an important ecological strategy for them to live in the deep-sea chemosynthetic ecosystems (Van Dover & Fry, 1994; Dubilier, Bergin & Lott, 2008). How the symbiosis is established and maintained has therefore been massively studied ever since while the immune recognition mechanisms yet was not well understood to date.

Bathymodiolinae mussels are one of the most common and dominant megafauna in deep-sea cold seeps and hydrothermal vents (Sibuet & Olu, 1998; Desbruyères et al., 2001; Van Dover et al., 2002; Laming, Gaudron & Duperron, 2018). As found, almost all of them can harbor γ-proteobacteria (mainly MOB and/or SOB) in the gill epithelial cells (also known as bacteriocytes) as their nutrition resources (Cavanaugh, Wirsen & Jannasch, 1992; Nelson, Hagen & Edwards, 1995; Duperron et al., 2006). It was found that Bathymodiolinae mussels could acquire their symbionts through horizontal transmission in all their life-span while the symbiosis mostly occurs in their juvenile stage (Fontanez & Cavanaugh, 2014; Wentrup et al., 2014). Further studies also discussed the diversity and specificity of the symbiont strains within the same species of Bathymodiolinae mussels in different colony distribution. Though the symbiont subpopulations seemed to vary with the environments, geographic distance and host development stage, the overall types of symbionts within a single mussel species were relatively conversed (Ikuta et al., 2016; Ansorge et al., 2019; Brzechffa & Goffredi, 2020; Ücker et al., 2020). It was therefore speculated that the Bathymodiolinae host can identify the symbiont by recognizing the microbe-associated molecular patterns (MAMPs) on the cell surface of symbionts such as sugars and glycoproteins. However, the lack of cultivable symbionts has hindered the characterization of symbiosis-related MAMPs in symbiotic MOB or SOB. On the other hand, pattern recognition receptors (PRRs) are known as the most critical molecules for the hosts to recognize symbiont MAMPs (Janeway & Medzhitov, 2002; Akira, Uematsu & Takeuchi, 2006). To date, multiple PRRs including peptidoglycan recognition proteins (PGRPs), Toll-like receptors (TLRs), lectins have been identified in other holobionts and found involved in the recognition of symbionts during the early stage of symbiosis (Janeway & Medzhitov, 2002; Chow et al., 2010; Chu & Mazmanian, 2013). To date, many efforts have been made to identify symbiosis-related PRRs in Bathymodiolinae mussels. For example, a decrease of PGRPs expression with the loss of symbionts was found in *Bathymodiolus azoricus*, suggesting its participation in the regulation of symbionts (Détrée et al., 2017). In a comparative study conducted with deep-sea mussels *B. azoricus* and shallow-water mussels *Mytilus galloprovincialis*, it was found that immune response of deep-sea mussels against *Vibrio* bacteria challenge was quicker than shallow-water mussels. Meanwhile, TLR2 and PGRPs could be vigorously modulated during the challenge of *Vibrio* bacteria (Martins et al., 2014). More recently, a global activation of host immune-related process during the deletion of symbionts was evidenced using proteomics and transcriptomics, which suggested an overall up-regulation of immune-related proteins and transcripts (Détrée et al., 2019). Despite the massive studies that have been conducted, the molecular mechanism beneath the mutual recognition and interaction between *Bathymodiolus* mussels and symbionts has remained still unclear.

*Gigantidas platifrons* (previously known as *Bathymodiolus platifrons*) are an endemic species that widely distributed in the cold seeps and hydrothermal vents of Northwest Pacific and merely host methanotrophs in their bacteriocytes (Barry et al., 2002). With the release of genomic information, *G. platifrons* has gradually been regarded as a model organism for studying the host-symbiont relationships in chemosynthetic ecosystem. The *G. platifrons* genome information showed that multiple PRRs including TLR13, PGRPs, immunoglobin domain and fibrinogen domain were remarkably expanded and highly expressed in gill tissue, implying their potential involvement in the immune recognition of symbionts (Sun et al., 2017). In addition, the transcriptome of *G. platifrons* revealed a higher expression of PRRs such as PGRPs, bactericidal permeability increasing protein and TLRs in the gill tissues in comparison with symbiont-free tissues such as foot and mantle tissues (Wong et al., 2015). Collectively, these studies suggested the crucial role of PRRs in the immune interaction between host mussels and symbionts while the identification of symbiosis-related PRRs remains difficult due to the lack of culturable symbionts. Recently, Chen et al. (2019) proposed with a modified MOB pull-down assay using purified symbiotic MOBs as bait to isolate PRRs that bind with symbionts while a LRR-domain containing gene (BpLRR-1) was identified as a putative intracellular recognition receptor for symbiotic MOBs in *G. platifrons* by binding with LPS. However, the overall number of identified PRRs is limited using MOB pull-down assay due to the purity and quantity of bacteria baits. In the meantime, a LPS pull-down assay was also performed in *G. platifrons* to identify symbiosis-related PRRs given that symbiotic MOB are γ-proteobacteria with lipopolysaccharide (LPS) structure in the cell wall. Consequently, a total of 208 proteins including BpLRR-1 and GpTLR13 were identified (Li et al., 2020). Among these genes, GpTLR13 was of interest given the crucial role of TLRs in symbiosis of multiple holobionts. TLRs are an important family of PRRs, which can sense the conserved molecular patterns of MAMPs and then activate immune response. Recently, TLRs are also reported in some Mollusca species. In Zhikong scallop *Chlamys farreri*, CfToll-1 was identified and its mRNA expression was up-regulated by LPS stimulation which showed a probable role in scallop immune response (Qiu et al., 2007). In addition, CfTLR, CfMyD88, CfTRAF6, CfIkB and CfNFkB were detected in *C. farreri* that indicated the presence of a MyD88-dependent TLR signaling pathway in scallop (Wang et al., 2011). In Pacific Oyster *Crassostrea gigas*, CgToll-1 was cloned and its expression pattern in hemolymph increased with the challenge of bacteria *Vibrio anguillarum* which suggested the key role of CgToll-1 in the immune defense against bacteria (Zhang, Li & Zhang, 2011). Besides, for mussels, two TLRs (McTLR2 and McTLR3) were identified and characterized in *Mytilus coruscus* and 23 TLRs were identified in *Mytilus galloprovincialis* (Toubiana et al., 2013; Li et al., 2019). Herein, we focused on GpTLR13 and thoroughly investigated its molecular characteristics. Moreover, the expression pattern of GpTLR13 with the loss of symbionts was examined by conducting a symbiont depletion assay under laboratory maintenance.

## Materials and Methods

### Mussels collection and symbiont depletion treatment

*G. platifrons* individuals were collected from the Formosa ridge cold seep of the South China Sea (22°06′N, 119°17′E; 1,113 m depth) using remotely operated vehicle (ROV) “Faxian” operated from R/V “Kexue” in 2017. Once the mussels were retrieved on the deck, gill tissues from 50 individuals were immediately dissected and then stored at −80 °C for pull-down assay or preserved in 75% ethanol at −20 °C after fixed in cold 4% paraformaldehyde overnight for ISH.

In addition, 30 *G. platifrons* mussels were transferred into a mussel cultivation system while the salinity was set to 34‰ and the temperature was kept at 3–4 °C for symbiont depletion treatment. During the experiment, the cultivation system was sheltered from light and no additional food was supplied but there might be some plankton in the seawater employed. Mussels were first acclimated for 48 h in the mussel cultivation system at atmospheric pressure to adapt the environmental change. Gill tissues from six mussels were dissected and stored at −80 °C as “0 day” group in symbiont depletion assay. Subsequently, ampicillin (Sangon Biotech, Shanghai, China) and streptomycin (Sangon Biotech, Shanghai, China) at a final concentration of 10 mg/L were supplement into aquarium and the seawater was changed every 3 days. Gill tissues from six mussels were sampled and pulled together every week during the treatment (7, 14, 21, 28 days post the treatment) and stored at −80 °C until use.

### The bioinformatics analysis of GpTLR13

The motif feature of GpTLR13 was annotated by SMART (http://smart.embl-heidelberg.de) and Pfam database. The presumed tertiary structure of GpTLR13 was predicted by Swiss-Model (http://swissmodel.expasy.org/interactive). The homologues of GpTLR13 were investigated by blastp algorithm in NCBI (https://blast.ncbi.nlm.nih.gov/Blast.cgi) and the similarity and identity among them were analyzed using Sequence Manipulation Suite (http://www.bioinformatics.org/sms/). The signal peptide of GpTLR13 was predicted by SignalP 5.0 program (http://www.cbs.dtu.dk/services/SignalP). The phylogenetic tree of TLRs was constructed based on multiple sequence alignment by the neighbor-joining method (NJ) using Mega X software. The reliability of branching was tested by bootstrap method which were replicated 1,000 times and the evolutionary distances were computed using the Poisson correction method.

### Gene cloning of GpTLR13

In this study, full-length cDNA of GpTLR13 coding region was cloned using cDNA template from gill tissues and primers designed according to *G. platifrons* genome information (Table 1). PCR amplification was conducted using ExTaq (Takara). In detail, the total reaction volume was 25 μL: 12.5 μL of exTaq, 1 μL of cDNA template, 0.5 μL of each TLR13_FL primer (Table 1) and 9.5 μL nuclease-free H2O. The PCR program was set as follows: 94 °C for 5 min, 35 cycles of 94 °C for 20 s, 50 °C for 30 s and 72 °C for 1 min, followed by the final extension at 72 °C for 10 min.

**Table 1:** Primers used in this study.

Primer	Sequence (5′-3′)	Application
TLR13_FL TLR13_RT	F: CTATTGAACAGGATGCCTTCTC	Gene cloning
	R: GTCGTCGCCTCCGTTCTT F: TCTTTCCCGATGAGTTTGTTGA R: CGCAGACGTTCGGGATAGG	qRT-PCR
Actin_RT	F: GACGAAGCCCAGGTAAAACG R: CTTAGTCATCATTTCTCTGTTGCCT	qRT-PCR
	
TLR13_POSITIVE_ISH	F: GAATCTGTCGGATTATAGTTGCTCA R: TAATACGACTCACTATAGGGATCCGTGACCAATCTGAAAGCC	ISH
TLR13_NEGATIVE_ISH	F: CGTGACCAATCTGAAAGCCCTG	ISH
	R: TAATACGACTCACTATAGGGATCGAATCTGTCGGATTATAGTTG	

The cloned gene product was separated by agarose gel electrophoresis and purified by Agarose Gel DNA Extraction Kit (Takara, Shiga, Japan). The purified cloned gene was connected to pMD19T vector (Sangon Biotech, Shanghai, China) for sequencing verification. The verified target gene was connected to the recombined adaptor through PCR reaction, and recombined into expression plasmid or other target plasmid through double enzyme digestion reaction, or used for qRT-PCR.

### qRT-PCR

The qRT-PCR was conducted to investigate the gene expression level of GpTLR13 during symbiont depletion using an Eppendorf Realplex4 thermocycler (Eppendorf, Hamburg, Germany) using SYBER Premix Ex Taq II (Tli RNaseH Plus) (Takara, Shiga, Japan). The reaction system was 10 µL containing 5 µL 2×SYBR Green PCR Master Mix, 0.2 µL of each TLR13_RT primer (Table 1), 0.2 µL Rox Reference Dye, 2 µL cDNA template and 2.4 µL DEPC water. The PCR program was set as follows: 94 °C for 5 min, 40 cycles of 94 °C for 5 s, 60 °C for 30 s, and 72 °C for 20 s. Meanwhile, additional melting curve analysis was performed to assess reaction specificity. Moreover, the PCR products have been subjected to both the agarose gel electrophoresis and sequencing to verify the specificity of GpTLR13 primers. Three biological replicates were employed for each group and gill tissue from two mussel individuals were pooled together as one biological replicate to minimize any influence due to the individual bias. Relative expression level of GpTLR13 was calculated using 2^−ΔΔCt^ method (Livak & Schmittgen, 2001) using actin gene as internal control. Data were analyzed by Kruskal–Wallis test followed by Dunn’s multiple comparison test when *p* < 0.05 using SPSS v19 to detect significant differences between groups.

### Prokaryotic expression and purification of GpTLR13

The cDNA fragment of GpTLR13 was connected to pET-30a vector (Takara, Shiga, Japan) with six His-tag, and then the recombined plasmid was transformed into Escherichia BL21(DE3) cell (Tiagen, Changping District, Beijing) for in vitro expression. During in vitro expression, targeted GpTLR13 protein was induced by IPTG (Sangon Biotech, Shanghai, China) for 24 h and purified by Ni-NTA affinity chromatography. The purity of recombined GpTLR13 protein was verified by SDS-PAGE, and the protein concentration was quantified by BCA kit (Beyotime Biotechnology, Shanghai, China).

### Surface plasmon resonance analysis

Since surface plasmon resonance (SPR) can quantitatively detect the interaction between proteins and other classes of biomolecules, it was performed to analyze the immune binging activity of GpTLR13 in *G. platifrons* according to the previously described method in present study (Lv et al., 2018). Firstly, the monoclonal antibody with His tags (Abclonal, Boston, MA, USA) was coupled to the CM5 sensor chip using Amine Coupling kit (GE Healthcare, Chicago, IL, USA) Then, a total of 40 µL GpTLR13 proteins (0.5 mg/mL) were loaded on the antibody-bound sensor chip reaching approximately 200 response units (RUs). A total of four PAMPs including LPS, peptidoglycan (PGN), lipid A, lipoteichoic acid (LTA) (1 mg/mL in PBS, Sigma-Aldrich) were supplemented into CM5 sensor chip at a speed of 10 µL/min and RUs values between TLR3 and four MAMPs were recorded during 2 min reactions. The binding activities between GpTLR13 and MAMPs were calculated using the Langmuir binding model and it was considered to have binding activity if RUs > 30.

### in situ hybridization (ISH) of GpTLR13

The synthesis of ISH probes and ISH assay were conducted using method previously described by Cheng et al. (2016). In brief, the DNA fragments of GpTLR13 gene were first amplified using specific primers pair (Table 1) as probe template. Digoxigenin-labeled ssRNA probes including antisense probe and sense probe were synthesized using DIG RNA Labeling kit (Roche, Basel, Switzerland). Meanwhile, gills stored in 75% ethanol were dehydrated and embedded with paraffin. Sections with 7 µm thickness were firstly pre-hybridized in hybridization buffer (50% formamide, 5×SSC, 50 μg/mL Heparin sodium, 0.5 mg/mL fish sperm DNA, and 0.01% Tween20) for 3 h at 37 °C, followed by hybridization for 12–16 h at 37 °C with 0.5 ng/µL probes in hybridization buffer. Afterward, the slides were washed in PBST and 4×SSC twice for 10 min each, and incubated with 2% BSA for 30 min. Then the slides were incubated with 1‰ anti-digoxigenin-AP Fab fragments in PBST for 4h. After washed by PBST, sections were incubated in development buffer pH 9.5 (100 mM Tris/HCl, 100 mM NaCl, 50 mM MgCl2) for 10 min and stained by BCIP/NBT Alkaline Phosphatase Color Development Kit (Sangon Biotech, Shanghai, China) for 6–12 h. The reaction was then terminated by water and imaged with microscopy after staining nucleic acids with 4′,6-diamidino-2-phenylindole (DAPI).

## Results

### Molecular characteristics of GpTLR13 protein

**Figure 1:**
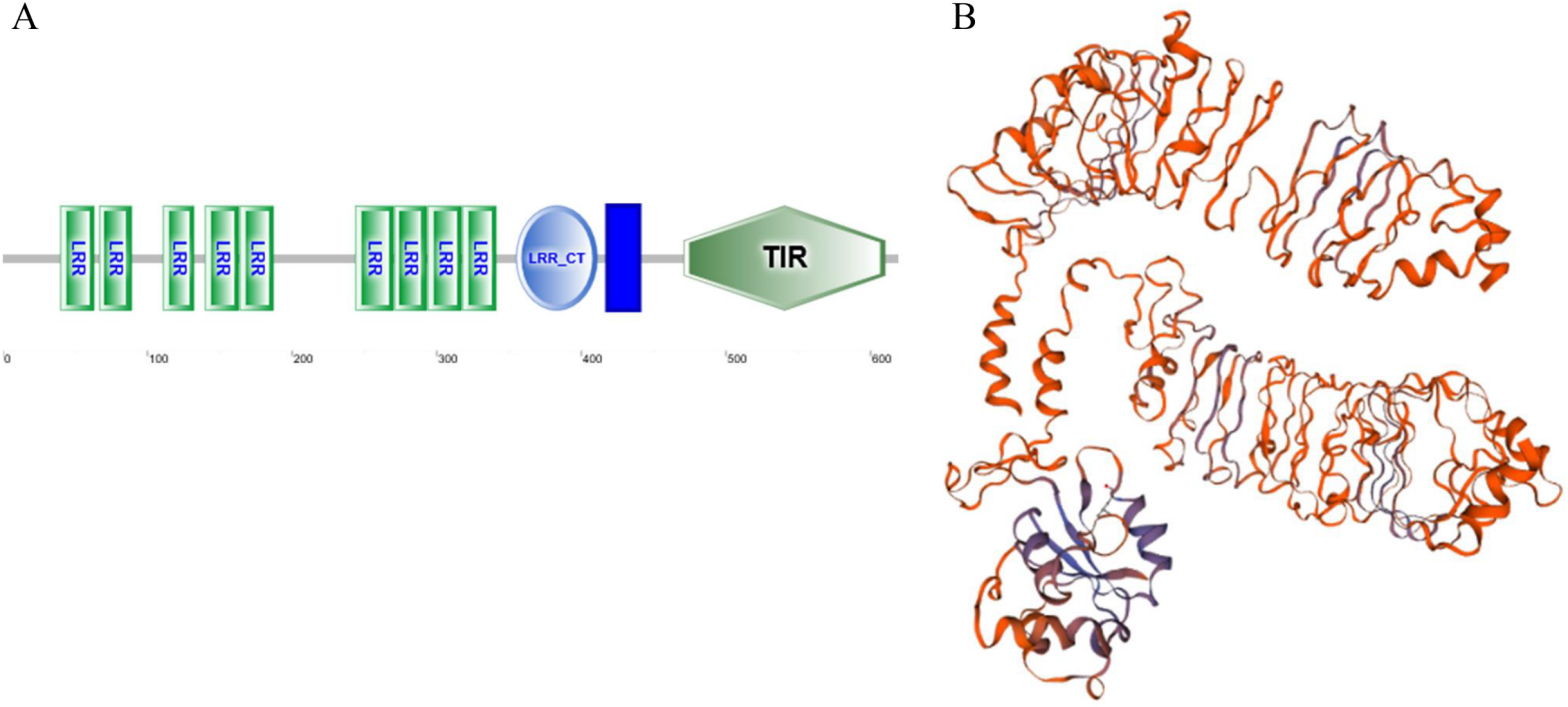
The protein structure of GpTLR13. (A) The protein domains of GpTLR13. A total of nine LRR domains, a LRR_CT domain, a TIR domain and a transmembrane region were observed. (B) The predicted three-dimensional structure of GpTLR13. A total of twenty α-helix and twenty-nine β-sheets were predicted.

**Figure 2:**
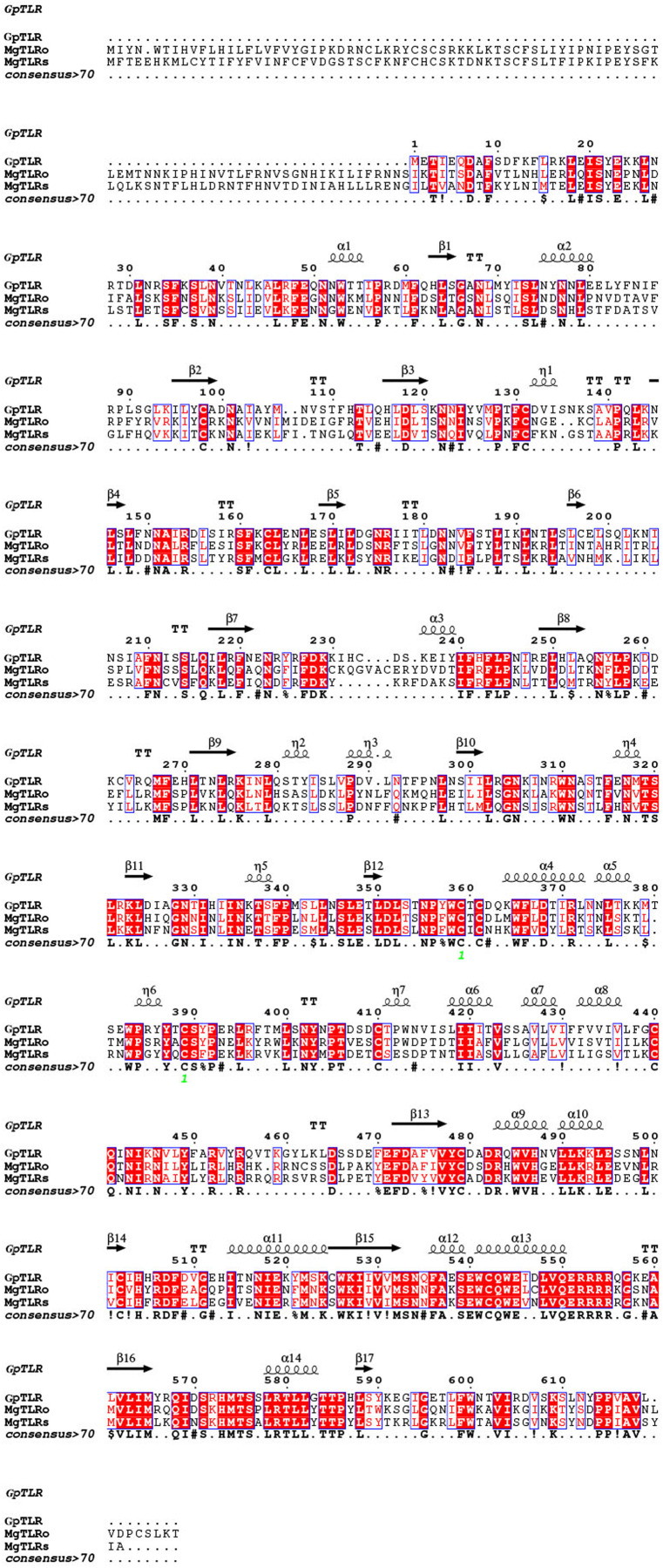
Amino acid sequences of GpTLR13 homologues aligned with MgTLRo and MgTLRs from *Mytilus galloprovincialis*.

**Figure 3:**
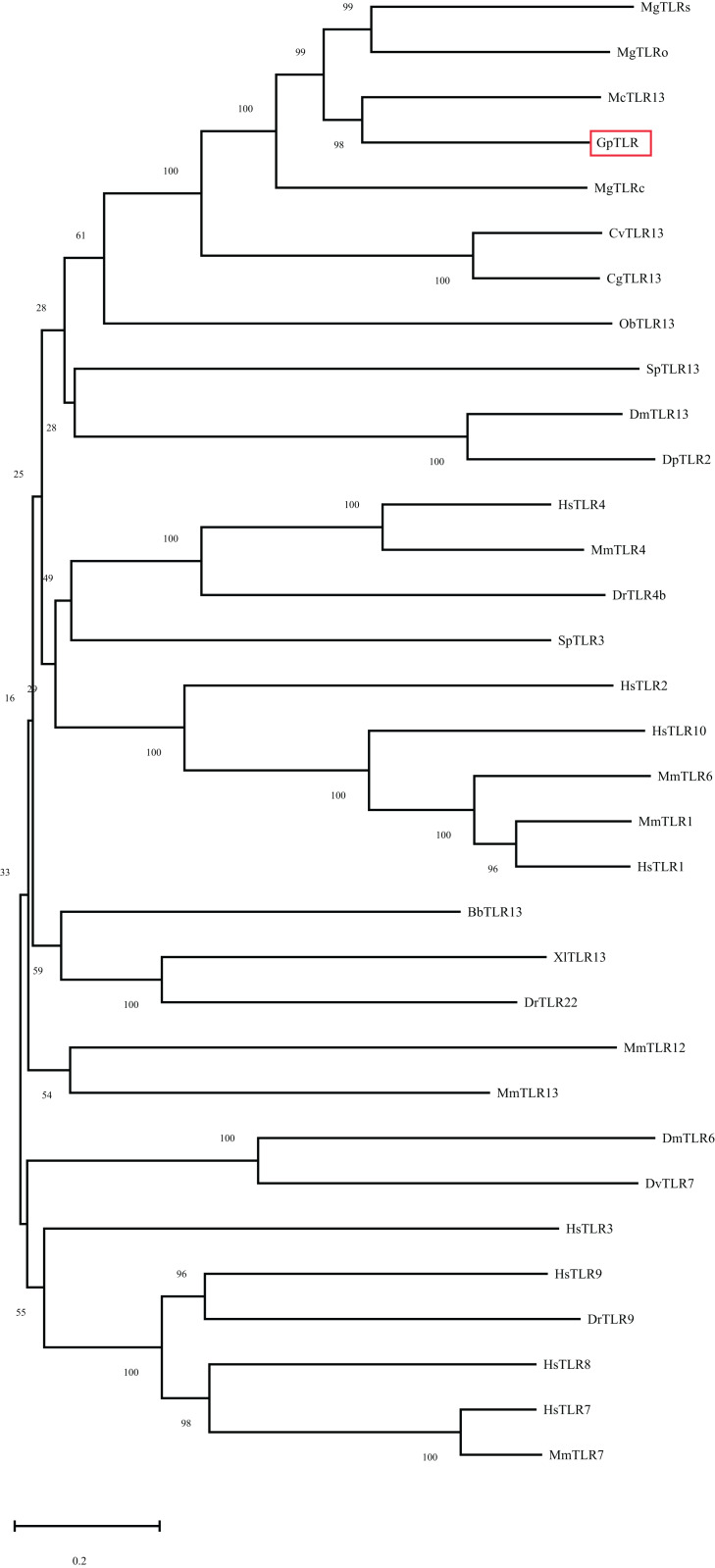
Phylogenetic tree of GpTLR13 built by neighbor-joining method based on the multiple sequence alignment. The reliability of the branching was tested by bootstrap resampling (1000 replicates). Red box indicates the GpTLR13 identified in the present study. The species and their accession numbers. are as follows: McTLR13 (*Mytilus coruscus*, CAC5384610.1), MgTLRs (*Mytilus galloprovincialis*, AGI05195.1), MgTLRo (*Mytilus galloprovincialis*, AGI05191.1), MgTLRc (*Mytilus galloprovincialis*, AFU48615.1), CgTLR13 (*Crassostrea gigas*, XP_034300167.1), CvTLR13 (*Crassostrea virginica*, XP_022312857.1), ObTLR13 (*Octopus bimaculoides*, XP_014771062.1), SpTLR13 (*Strongylocentrotus purpuratus*, XP_030850978.1), DmTLR13 (*Drosophila mojavensis*, XP_002008252.2), DpTLR2 (*Drosophila persimilis*, XP_026845412.1), HsTLR4 (*Homo sapiens*, NP_612567.1), MmTLR4 (*Mus musculus*, NP_067272.1), DrTLR4b (*Danio rerio*, NP_997978.2), SpTLR3 (*Strongylocentrotus purpuratus*, XP_011681200.1), HsTLR2 (*Homo sapiens*, NP_001305725.1), HsTLR10 (*Homo sapiens*, NP_001182036.1), MmTLR6 (*Mus musculus*, NP_001371100.1), MmTLR1 (*Mus musculus*, AAG35062.1), HsTLR1 (*Homo sapiens*, AAH89403.1), BbTLR13 (*Branchiostoma belcheri*, XP_019638324.1), XlTLR13 (*Xenopus laevis*, XP_018106869.1), DrTLR22 (*Danio rerio*, NP_001122147.2), MmTLR12 (*Mus musculus*, NP_991392.1), MmTLR13 (*Mus musculus*, NP_991389.1), DmTLR6 (*Drosophila mojavensis*, XP_002007724.1), DvTLR7 (*Drosophila virilis*, XP_002049095.1), HsTLR3 (*Homo sapiens*, NP_003256.1), HsTLR9 (*Homo sapiens*, NP_059138.1), DrTLR9 (*Danio rerio*, NP_001124066.1), HsTLR8 (*Homo sapiens*, NP_057694.2), HsTLR7 (*Homo sapiens*, NP_057646.1), MmTLR7 (*Mus musculus*, NP_001277684.1). The scale bare refers to 20% sequence variation.

As a candidate PRR, a total of eight peptides of GpTLR13 were identified by LC-MS/MS (Table 2). The amino acid sequence of GpTLR13 protein was obtained and annotated by Pfam database. The results showed that the full length of GpTLR13 coding region was 1,860 bp and the open reading frame encodes a polypeptide with molecular weight approximately 72.58 kDa (Supplemental File 1). GpTLR13 protein contains nine LRR domains, a LRR_CT domain, a TIR domain and a transmembrane region annotated by SMART (Fig. 1A). The tertiary structure of GpTLR13 protein predicted by Swiss-model consisted of twenty α-helix and twenty-nine β-sheets (Fig. 1B). To investigate the evolution relationship of GpTLR13 with other TLRs, the homologues of GpTLR13 were aligned by blastp algorithm in NCBI and the similarity and identity among them were investigated by Sequence Manipulation Suite (Table 3). The amino acid sequences of GpTLR13 in *G. platifrons* were similar to McTLR13 (CAC5384610.1, 56.5% similarity) from *Mytilus coruscus*, MgTLRo (GenBank accession No. AGI05191.1, 40.6%), MgTLRs (AGI05195.1, 39.9%) and MgTLRc (AFU48615.1, 50.1%) from *Mytilus galloprovincialis*, CvTLR13 (XP_022312857.1, 43.7%) from *Crassostrea virginica*, CgTLR13 (XP_034300167.1, 48.3%) from *Crassostrea gigas*, and ObTLR13 (XP_014771062.1, 33.5%) from *Octopus bimaculoides*. Among them, it was most similar to that of MgTLRo and MgTLRs from *Mytilus galloprovincialis* (Fig. 2). The naming of the GpTLR gene was based on the amino acid sequence similarity with other TLRs and their clustering in phylogenetic tree (Fig. 3). We named this gene GpTLR13 since GpTLR identified in present study was more similar to McTLR13 and clustered in the phylogenetic tree with it.

**Table 2:** Protein fragments of GpTLR13 identified by LC-MS/MS.

Start-End	Mr (expt)	Mr (calc)	Sequence
26-33	1,000.5636	1,000.5414	K.LNRTDLNR.S
26-33	1,002.5130	1,002.5094	K.LNRTDLNR.S + 2 Deamidated (NQ)
610-619	1,072.5634	1,072.5804	K.SLNYPPVAVL.- + Deamidated (NQ)
610-619	1,072.5634	1,072.5804	K.SLNYPPVAVL.- + Deamidated (NQ)
610-619	1,072.5634	1,072.5804	K.SLNYPPVAVL.- + Deamidated (NQ)
610-619	1,072.5634	1,072.5804	K.SLNYPPVAVL.- + Deamidated (NQ)
484-492	1,136.6004	1,136.6342	R.QWVHNVLLK.K + Deamidated (NQ)
484-492	1,136.6004	1,136.6342	R.QWVHNVLLK.K + Deamidated (NQ)

**Table 3:** Similarity of GpTLR13 with its homologues aligned from the protein sequence.

Proteins	Organisms	Accession number	Similarity %	Identify %
McTLR13	*Mytilus coruscus*	CAC5384610.1	56.5	44.1
MgTLRo	*Mytilus galloprovincialis*	AGI05191.1	50.1	40.6
MgTLRs	*Mytilus galloprovincialis*	AGI05195.1	49.9	38.9
MgTLRc	*Mytilus galloprovincialis*	AFU48615.1	50.1	37.5
CvTLR13	*Crassostrea virginica*	XP_022312857.1	43.7	29.3
CgTLR13	*Crassostrea gigas*	XP_034300167.1	48.3	33.4
ObTLR13	*Octopus bimaculoides*	XP_014771062.1	33.5	21.8

### The binding pattern of GpTLR13 protein

**Figure 4:**
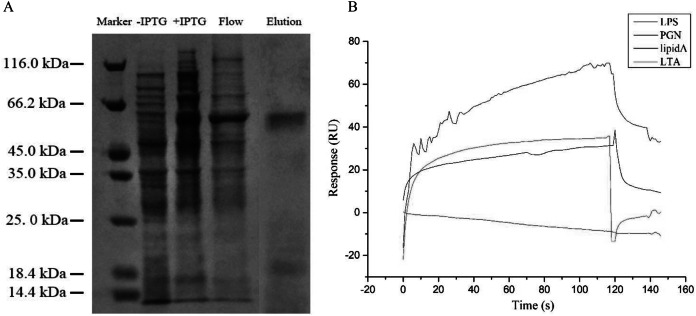
Binding pattern of GpTLR13 with MAMPs. (A) Recombined GpTLR13 with His tags that were expressed in *E.coil* BL21 cells were purified by Ni-NTA affinity chromatography and verified by SDS-PAGE. During protein purify, besides the purified GpTLR13 protein, lysates of BL21 cell without IPTG induction (“- IPTG”) and with IPTG induction for 24h at 20 °C (“+ IPTG”) were also loaded onto Ni-NTA column (“Flow”). Then purified GpTLR13 protein was separated by SDS-PAGE and stained with coomassie blue. (B) The binding activities of GpTLR13 against diverse MAMPs including LPS, PGN, lipid A and LTA by SPR assay.

After IPTG induction, the recombined GpTLR13 of which some amino were removed from C- and N-terminals during the recombination with Pet-30a vector was purified by Ni-NTA affinity chromatography and isolated by SDS-PAGE (Fig. 4A). A distinct band with molecular weight of ~61.45 kDa was obtained. Then the binding activities of GpTLR13 against diverse MAMPs were analyzed by SPR assay (Fig. 4B). As a result, GpTLR13 had a significant binding activity with LPS which was the main contents on the membrane of Gram-negative bacteria as it responded quickly and peaked approximately at 38 RUs. Meanwhile, GpTLR13 also can bind with lipid A and LTA, which was an important part of LPS and the important surface antigen of Gram-positive bacteria, respectively. Among them, the binding activity with lipid A was highest, reaching 70 RUs. However, negative SPR signal was generated when GpTLR13 was incubated with PGN which was from cell walls of Gram-positive bacteria.

### Expression pattern of GpTLR13 gene during symbiont depletion

**Figure 5:**
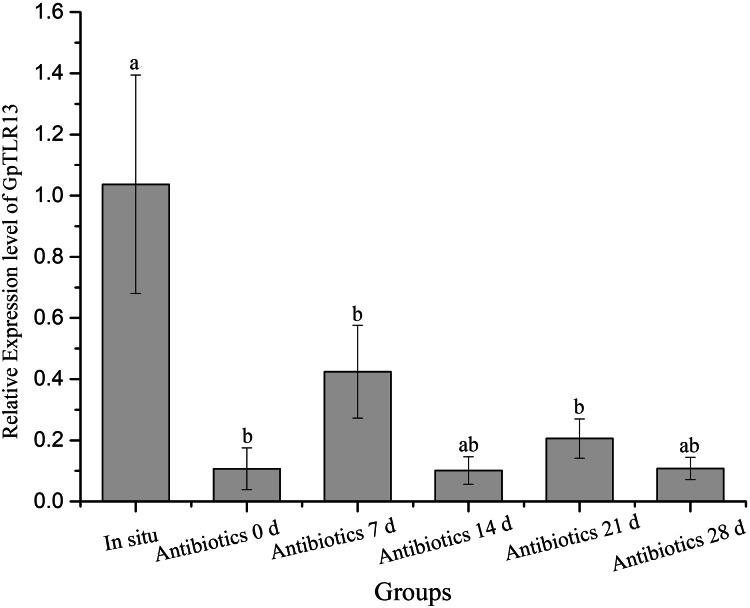
Relative expression level of GpTLR13 in the gills of *G. platifrons* during antibiotics treatment. “a”, “b” represent significant differences between different groups.

The expression pattern of GpTLR13 transcripts in *G. platifrons* gills during symbiont-depletion assay was then surveyed by qRT-PCR (Fig. 5). The abundance of symbionts during antibiotics treatment was quantified and reported in Chen et al. (2019) which showed a significantly decrease of symbionts during 28-days antibiotics treatment. The qRT-PCR results showed that after the treatment of antibiotics, the relatively expression level of GpTLR13 in *G. platifrons* gills decreased rapidly with the loss of symbionts. In detail, the expression level had reduced to 0.1-fold of in situ after 2 days of acclimation, and reached 0.08-fold of in situ after 28 days of antibiotics treatment.

### mRNA distribution of GpTLR13 in gill tissues

**Figure 6:**
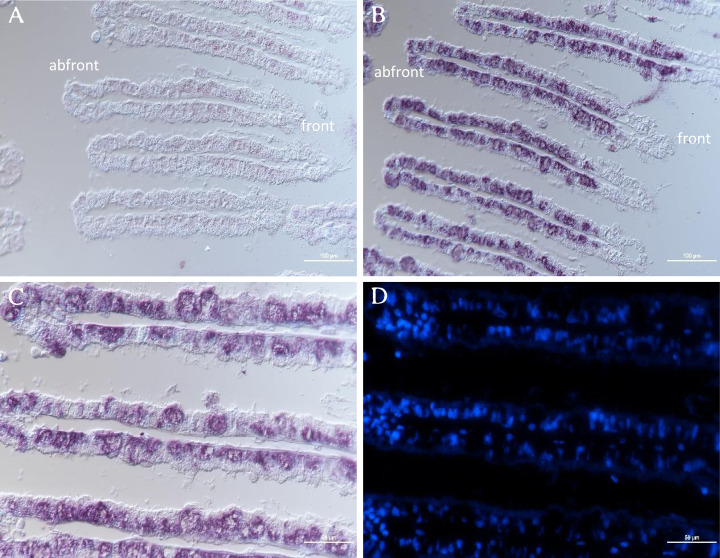
ISH of GpTLR13 in the gill of *G. platifrons*. **** (A) ISH target to GpTLR13 sense mRNA (negative control). Each gill filament has a ciliated frontal edge and a bacteria-colonized abfrontal edge. (B–C) ISH target to GpTLR13 antisense mRNA (positive signal). The different regions of the same slide were showed in B and C. (D) FISH with DAPI. D was the same region as C. Scale bar: A–B. 100 μm; C–D. 50 μm.

The distribution of GpTLR13 mRNA in gill tissue of *G. platifrons* was then surveyed by ISH. As showed in Figs. 6A and 6B, each gill filament has a ciliated frontal edge and a non-ciliated abfrontal edge by cross-sectioning. The cells on the ciliated frontal edge are not colonized by symbionts and other gill cells including abfrontal edge which lacked microvilli are bacteriocyte. As a result, the GpTLR13 mRNA signals were found in the bacteriocytes of gills instead of the ciliated cells (Figs. 6A–6C). After co-localization with the nuclei of gill cells, majority of GpTLR13 mRNA was found expressed at the nuclei region of the bacteriocytes (Fig. 6D).

## Discussion

Symbiosis between microorganisms and multicellular organisms could be widely observed across plants or animals. In some circumstances, the symbiosis could be species-specific where host could discriminate the symbiotic bacteria from the non-symbiotic bacteria in surrounding environments before the symbiotic associations are established. During symbiosis establishment, immune receptors of hosts such as PRRs are required to promote the colonization of symbionts. PRRs are known as an important part of the immune system that plays a crucial role in symbiotic recognition. Generally, PRRs could bind with the MAMPs on the surface of symbionts such as LPS, LTA, PGN and flagellin and activate the downstream signaling pathways that generate a series of immune responses through signaling transduction cascade (Janeway & Medzhitov, 2002). In our study, to get a better understanding of how host mussels recognize symbiotic MOBs, we performed a cascade of experiments aimed at screening symbiosis-related PRRs and function analysis. Due to the limitation of MOB pull-down and the fact that symbiotic MOBs are γ-proteobacteria with lipopolysaccharide (LPS) structure in the cell wall, we firstly isolated 208 PRRs potentially binding with LPS including GpTLR13 protein in *G. platifrons* gills using pull-down with LPS as bait. Based on the previously reported genome and transcriptome information of *G. platifrons*, GpTLR13 was greatly expanded and highly expressed in gill tissue, implying its potential role in symbiosis (Wong et al., 2015; Sun et al., 2017). Meanwhile, recent study reported that TLRs were significantly enriched in bacteriocyte transcriptomes in *G. platifrons* by profiling the transcriptomes of the gill, bacteriocyte, and symbiont, respectively, which confirmed the important role of TLRs in symbiosis (Wang et al., 2021). Therefore, the molecular character and immune function of GpTLR13 were further analyzed to address its role in symbiosis.

TLRs are evolutionarily conserved PRRs that play important roles in immune defense by recognizing conversed structures in microorganisms and transducing signals to trigger innate immune response (Kawai & Akira, 2010). Molecular and functional characterization of TLRs have been reported in other marine bivalve species, including mussels, clams, oysters, and scallop (Gerdol & Venier, 2015; Ren et al., 2016; Xing et al., 2017; Peng et al., 2020; Ren et al., 2020). Besides, TLRs also play vital roles in symbiotic mutual interaction (Kubinak & Round, 2012). TLR13 is a member of the TLR11 family, which is an important part of innate immune system. The presence and conversed structure characterization of TLR13 have been revealed in oyster and scallop (Xing et al., 2017; Peng et al., 2020). In our study, we identified a total of eight peptides of GpTLR13 by LC-MS/MS, which demonstrated the reliability of pull-down results. SMART analysis showed that GpTLR13 possessed the typical structures of the TLR13 proteins, which was composed of extracellular region including nine LRR motifs and a LRR-CT motif, a cysteine-rich transmembrane region and cytoplasmic region including a TIR motif. In detail, LRR motif in the extracellular region took part in the recognition of pathogen effectors while TIR motif in the cytoplasmic region can interact with TIR domain-containing adaptor proteins to initiate signaling, which was the core element in downstream signaling (Akira & Takeda, 2004). Moreover, the three-dimensional structure of GpTLR13 was consistent with the typical structure of TLR13 proteins (Song et al., 2015). Collective results showed that the structures of GpTLR13 were highly conversed which suggested its conversed microbial recognition function and the potential ability of symbiotic MOBs recognition.

In general, TLR13 was known as an endosomal TLR, which can play a role in recognition of microbes only after their phagocytosis and specifically recognize conversed bacterial 23S rDNA sequences. Nevertheless, it was speculated that TLR13 can also recognize MAMPs including LPS, LTA, PGN, poly(I-C) in previous study (Shi et al., 2009; Tang et al., 2020). However, the exact ligand of TLR13 in mussels remains unclear now. Herein, to verify the reliability of LPS pull-down results and the potential ligand of GpTLR13, we investigated the binding activities of GpTLR13 to various PAMPs. The results showed that GpTLR13 can bind with LPS, lipid A and LTA while had not the binding activity to PGN which was the typical PAMP of Gram-positive bacteria. LPS are the characteristic PAMP located on the outer membrane of Gram-negative bacteria, which are composed of three sections: hydrophobic lipid A, hydrophilic core oligosaccharide and hydrophilic O-specific side chain. Among them, lipid A is responsible for the toxic properties and biological activities of bacteria endotoxin. GpTLR13 had also a relatively high binding activity to LTA which was widespread in the cell wall of Gram-positive bacteria, which may indicate GpTLR13 retain conversed ligand recognition and binding ability. Taken together, its specific binding pattern implied that GpTLR13 recognize symbiotic MOBs by binding with LPS structure in the plasma membrane. However, we have not addressed the direct interaction between GpTLR13 and symbiotic MOBs that due to the lack of suitable antibody of GpTLR13 and pure bacteria baits. It is well documented that a close connection between the expression of TLR13 and the abundance of symbionts was showed in *G. platifrons* and *Bathymodiolus manusensis* (Zheng et al., 2017; Wang et al., 2019). To further determinate if GpTLR13 took part in symbiosis, we also investigated the expression level of GpTLR13 during antibiotics treatment and its expression pattern in gill tissue. For symbiont depletion assay, we have tried our best to mimic the parameters in deep-sea (except for the oxygen concentration and pressure) to reduce the interference of environmental changes on the expression of GpTLR13 and made the results more convincing. And we have also surveyed the alternations of caspase 3 activity, total antioxidant capacity and SOD activity of Mytilus mussels during a 3-day antibiotics treatment yet found no significant alternation. Therefore, we believed that the decrease of GpTLR13 transcripts was most likely resulted from symbiont depletion. Though no data showed the depletion of symbionts with antibiotics treatment in present study, previous work from our team by Chen et al. (2019) has confirmed that symbionts could be effectively depleted using antibiotics treatment. Here, through a co-analysis with previous data, a remarkable negative correlation between the expression of GpTLR13 and the abundance of symbionts and its exclusive expression pattern in bacteriocytes of gills confirmed the participation of GpTLR13 in symbiosis.

Unlike other members of TLRs family, few studies about TLR13 signaling pathways and functions in invertebrates were reported up to date. Previous study found that TLR13 played roles in innate immunity in clams *Cyclina sinensis* through the TLR13-MyD88-NF-kB signaling pathway (Ren et al., 2017). In brief, after binding with PAMPs, TLR13 recruited the Myeloid Differentiation Factor 88 (MyD88) which was a vital adaptor protein in the TLRs signaling pathway. Subsequently, the nuclear factor-kappa B (NF-kB) signaling pathway was activated and ultimately triggered the immune responses. In addition, the high expression of MyD88 gene after the acclimation in atmosphere pressure also suggested an induction on TLR signaling pathway (Barros et al., 2015). In the present study, A TIR motif was found in GpTLR13 protein, which could bind with the TIR motif of MyD88. Given these results, we assumed that GpTLR13 participated in immune recognition of symbiotic MOBs in *G. platifrons* by binding with LPS and triggering the immune responses through MyD88-dependent NF-kB signaling pathway. However, the detail of the signaling pathway requires further study to support this hypothesis.

## Conclusion

In present study, GpTLR13 was isolated and identified by LPS pull-down assay that suggested its potential role in the symbiosis between *G. platifrons* and MOBs. The predicted structure characteristics and the MAMPs binding pattern showed that GpTLR13 possessed the conservative structure of conventional TLR13 and could recognize LPS structure by binding with lipid A. These results further hinted that GpTLR13 was an important symbiotic-related PRR. The expression levels during symbiont depletion based on the successful mussel cultivation in the lab. and specific expression pattern in the bacteriocytes of gills verified that GpTLR13 took part in immune recognition of symbiotic MOBs. Overall, although we lacked more direct evidence to support that GpTLR13 could bind with symbiotic MOBs, our results still indicated that GpTLR13 participated in symbiosis as an immune recognition receptor. Further studies should clarify the downstream signaling pathway of GpTLR13. As for now, our study also provides new clues for understanding molecular mechanisms underlying the interaction between host mussels and symbionts.

## Supplemental Information

10.7717/peerj.11282/supp-1 Supplemental Information 1 Nucleotide and deduced amino acid sequence of GpTLR13.

10.7717/peerj.11282/supp-2 Supplemental Information 2 The raw data of qRT-PCR.

10.7717/peerj.11282/supp-3 Supplemental Information 3 Protein sequence of GpTLR13.

10.7717/peerj.11282/supp-4 Supplemental Information 4 Coding sequence of GpTLR13 gene.

## Additional Information and Declarations

### Data Availability

The following information was supplied regarding data availability: The raw measurements are available in the Supplementary Files. The DNA and protein sequences are available at GenBank: MW400609 and in the Supplementary Files.
