# Supplementary material for: A Toll-like receptor identified in Gigantidas platifrons and its potential role in the immune recognition of endosymbiotic methane oxidation bacteria
Source: PeerJ. 2021 Apr 30;9:e11282. doi: 10.7717/peerj.11282 (PMC8092104; doi:10.7717/peerj.11282)
Supplement: Supplemental Information 1 [file peerj-09-11282-s001.docx]

1 M E T I E Q D A F S D F K F L R K L E I

1 ATGGAAACTATTGAACAGGATGCCTTCTCAGATTTCAAATTTCTTAGAAAATTAGAAATA

21 S Y E K K L N R T D L N R S F K S L N V

61 AGTTACGAGAAAAAATTGAATAGGACCGATTTAAACAGATCCTTTAAAAGCTTGAATGTT

41 T N L K A L R F E Q N N W T T I P R D M

121 ACCAACCTGAAGGCTTTAAGGTTTGAACAAAACAATTGGACAACAATACCACGTGATATG

61 F Q H L S G A N L M Y I S L N Y N N L E

181 TTTCAACATTTATCAGGAGCCAATCTTATGTATATATCTCTCAATTACAATAATTTGGAG

81 E L Y F N I F R P L S G L K I L Y C A D

241 GAACTTTATTTTAATATATTTAGACCGCTTAGTGGATTAAAGATACTTTATTGTGCAGAC

101 N A I A Y M N V S T F H T L Q H L D L S

301 AACGCAATTGCTTATATGAATGTAAGTACATTTCACACATTGCAACATCTAGATCTTTCC

121 K N N I Y V M P T F C D V I S N K S A V

361 AAAAATAACATTTACGTCATGCCTACGTTTTGTGACGTCATCAGTAACAAAAGTGCTGTT

141 P Q L K N L S L F N N A I R D I S I R S

421 CCACAACTGAAGAACTTATCGCTGTTTAACAATGCTATTCGAGACATTTCGATTCGTTCA

161 F K C L E N L E S L I L D G N R I I T L

481 TTCAAATGCTTGGAAAATTTAGAATCACTCATTTTGGATGGAAATCGCATTATAACGTTA

181 D N N V F S T L I K L N T L S L C E L S

541 GATAATAATGTTTTTTCCACTTTGATTAAATTAAATACATTGTCGTTGTGTGAACTGTCA

201 Q L K N I N S I A F N I S S L Q I L R F

601 CAACTTAAGAACATAAATAGTATAGCATTCAACATTTCATCCTTACAAATATTACGTTTT

221 N E N R Y R F D K K I H C D S K E I Y I

661 AACGAAAATAGATATCGTTTTGATAAAAAGATTCATTGCGATTCTAAAGAAATTTATATT

241 F H F L P N I R E L H L A Q N Y L P K D

721 TTTCATTTTTTACCAAATATAAGGGAACTTCACCTGGCTCAAAACTATTTACCAAAAGAT

261 D K C V R Q M F E H L T N L R K I N L Q

781 GATAAATGTGTTCGTCAAATGTTTGAACATCTCACAAATTTAAGAAAAATTAATCTTCAA

281 S T Y I S L V P D V L N T F P N L N S I

841 TCTACTTATATCAGCTTGGTACCAGACGTACTTAACACATTCCCGAATTTAAACTCGATA

301 I L R G N K I N R W N A S T F E N M T S

901 ATTTTACGTGGAAACAAAATAAACCGATGGAATGCATCAACATTTGAAAATATGACGTCA

321 L R K L D I A G N T I H I I N K T S F P

961 CTGCGAAAATTAGATATTGCTGGAAATACCATTCATATAATAAACAAAACATCTTTCCCG

341 M S L L N S L E T L D L S T N P Y W C T

1021 ATGAGTTTGTTGAATAGTTTGGAAACACTTGACCTTTCGACCAATCCTTATTGGTGTACC

361 C D Q K W F L D T I R L N N L T K K M T

1081 TGTGATCAAAAATGGTTTCTAGATACAATAAGATTAAACAATTTGACAAAGAAGATGACA

381 S E W P R Y Y T C S Y P E R L R F T M L

1141 TCCGAATGGCCAAGATATTATACTTGTTCCTATCCCGAACGTCTGCGGTTTACCATGTTG

401 S N Y N P T D S D C T P W N V I S L I I

1201 TCCAACTACAATCCAACCGATTCAGACTGCACACCGTGGAACGTCATATCTCTCATCATT

421 I T V S S A V L V I F F V V I V L F G C

1261 ATAACTGTGTCGTCTGCCGTGTTGGTTATATTTTTTGTGGTCATAGTGCTGTTTGGATGT

441 Q I N I K N V L Y F A R V Y R Q V T K G

1321 CAAATTAATATCAAAAACGTATTATACTTCGCTCGTGTATACCGACAGGTGACAAAAGGA

461 Y L K L D S S D E F E F D A F V V Y C D

1381 TATCTTAAATTAGATTCATCAGACGAGTTTGAATTTGACGCCTTTGTTGTGTATTGTGAC

481 A D R Q W V H N V L L K K L E S S N L N

1441 GCTGATCGTCAGTGGGTTCATAATGTCTTGTTAAAGAAATTAGAGAGTTCTAATTTGAAT

501 I C I H H R D F D V G E H I T N N I E K

1501 ATATGCATTCACCATCGCGATTTTGATGTCGGAGAGCATATTACTAACAATATAGAGAAA

521 Y M S K C W K I I V V M S N Q F A E S E

1561 TATATGAGCAAATGTTGGAAAATAATAGTTGTTATGTCAAATCAATTCGCTGAAAGTGAA

541 W C Q W E I D L V Q E R R R R Q G K E A

1621 TGGTGCCAATGGGAAATTGACCTTGTCCAAGAACGGAGGCGACGACAGGGAAAGGAAGCA

561 L V L I M Y R Q I D S R H M T S S L R T

1681 TTAGTCCTAATTATGTATCGTCAGATAGACTCGCGACATATGACTAGTTCTCTGCGAACA

581 L L G T T P H L S Y K E G I G E T L F W

1741 TTACTAGGTACAACACCGCATCTCTCTTATAAAGAAGGAATTGGGGAAACTCTTTTTTGG

601 N T V I R D V S K S L N Y P P V A V L *

1801 AATACAGTTATCAGGGATGTGAGCAAGTCACTAAATTATCCACCAGTAGCTGTTTTATAA
